# The Regulation of Osteogenesis Using Electroactive Polypyrrole Films

**DOI:** 10.3390/polym8070258

**Published:** 2016-07-13

**Authors:** Chuan Li, Yi-Ting Hsu, Wei-Wen Hu

**Affiliations:** 1 Department of Biomedical Engineering, Yang-Ming University, Taipei 11221, Taiwan; 2 Centre for Biomedical Cell Engineering, National Central University, Zhongli District, Taoyuan City 32001, Taiwan; 3 Department of Chemical and Materials Engineering, National Central University, Zhongli District, Taoyuan City 32001, Taiwan; yearnfor530@hotmail.com

**Keywords:** polypyrrole, osteogenesis, conductive polymer, electroactive, bone marrow stromal cells, mineralization

## Abstract

To evaluate the effect of electrical conductivity of biomaterials on osteogenesis, polypyrrole (PPy) was fabricated by oxidative chemical polymerization as substrates for cell culture. Through adjusting the concentrations of monomer and initiator, polypyrrole films with different electrical conductivities were fabricated. These fabricated polypyrrole films are transparent enough for easy optical microscopy. Fourier transform infrared spectroscopy, X-ray spectroscopy and four-point probe were used to assess the microstructures, surface chemical compositions and electrical sheet resistance of films, respectively. Results indicate that higher monomer and initiator concentration leads to highly-branched PPy chains and thus promotes the electron mobility and electrical conductivity. Selected polypyrrole films then were applied for culturing rat bone marrow stromal cells. Cell viability and mineralization assays reveal that not only these films are biocompatible, but also capable of enhancing the calcium deposition into the extra cellular matrix by the differentiated cells.

## 1. Introduction

Wolff‘s law is a golden rule for bone regeneration, which states that induced mineralization makes bones more resistant to mechanical loads. It was found that stress-induced stains may generate an electrical potential along the collagen fibers in bones to stimulate osteogenesis [1]. In other words, the bone regeneration may be due to the piezoelectric effect under which the electrical cue is generated by the external mechanical loads on bones. Therefore, electric stimulations are considered as physical signals to promote osteogenesis of stem or progenitor cells [2,3].

To simulate electrical cues generated by ECM during osteogenesis, substrate-mediated direct current field (DCF) is developed, in which the substrate for cell culture is electrically conductive and electrodes are placed outside the culture media. An immediate question for substrate-mediated DCF is the selection of materials for substrates. For the purpose of electric conducive, metals are the top choice but their biocompatibility is a major concern [4,5,6]. Moreover, the high density of current can damage cells during stimulation and the corrosion of metals in culture media can lead to the release of cytotoxic debris [7]. The next choice therefore is averted to polymers or polymeric composites. For example, multi-walled carbon nanotubes (MWCNTs) have been added to polymers to prepare electroactive scaffolds [8,9]. Polypyrrole (PPy) doped by various materials also can demonstrate enhanced conductivity [10,11,12]. However, these conductive polymers were opaque and unsuitable for optical microscopy. Fluorescent staining and scanning electron microscopy (SEM) were employed for observation; however, continuous monitoring cellular activities become impossible by these end-point analyses.

Therefore, transparent and conductive PPy films by chemical oxidation polymerization were prepared in this study. These films were biocompatible for cell culture and transparent and for optical microscopy. Through the control of monomer to initiator concentration ratio, the electrical conductivity of PPy films can be adjusted. Many studies indicate that electroactive surfaces of conductive polymers may increase intercellular communication to promote cell proliferation and adhesion [13,14,15,16]. Whether fabricated PPy films possess such capacity remains to be assessed. Our results demonstrate that the PPy films’ surface is highly electroactive, which is able to expedite the cell differentiation. These films’ properties suggest that the fabricated PPy thin films are potentially suitable for surface coating on medical implants in orthopedic applications.

## 2. Materials and Methods

### 2.1. Materials

Ammonium persulfate (APS) and pyrrole (Py) were purchased from Showa (Tokyo, Japan) and Acros (Geel, Belgium), respectively. Dulbecco’s modified Eagle medium (D-MEM), fetal bovine serum (FBS), and trypsin–EDTA were obtained from Biowest (Nuaillé, France). Triton X-100, dexamethasone, 2-phospho-L-ascorbic acid trisodium salt, β-glycerophosphate disodium salt hydrate, and glutaraldehyde were purchased from Sigma-Aldrich (St. Louis, MO, USA).

### 2.2. The Preparation of PPy Films

Polystyrene (PS) made petri dish (φ 35 mm in diameter, Nunc, Penfield, NY, USA) was used as the substrate for PPy films deposition. Pyrrole and APS aqueous solutions with different concentrations were gently mixed in petri dishes (2 mL) for 15 min at room temperature. The deposited films were washed by deionized water and dried in oven.

### 2.3. Fourier Transform Infrared (FTIR) Spectroscopy

To determine the structures of the deposited PPy films, attenuated total reflection-Fourier transform infrared (ATR-FTIR) spectrometer (Spectrum 100, Perkin Elmer, Waltham, MA, USA) was used with resolution of 1 cm^−1^. The range of spectrum is set between 400 to 3600 cm^−1^ and transmission mode is used for detections.

### 2.4. Scanning Electron Microscopy (SEM)

Surface morphologies of PPy films were observed by scanning electron microscopy (SEM, 3500N, Hitachi, Tokyo, Japan). Gold was plated on the films’ surface by sputtering before examination. The film thickness was measured from the cross sections by cyrofracturing of films in liquid nitrogen.

### 2.5. Four Point Probe Analysis

Four point probe (EverBeing, Hsinchu, Taiwan) was used to determine the sheet resistance of PPy film. Twenty spots were randomly selected in each sample to measure the spatial variation of the sheet resistance. The electrical conductivity of PPy films then was calculated based on the thickness measured by SEM.

### 2.6. X-Ray Photon-Electron Spectrometry (XPS)

Surface chemical analysis of PPy films was carried out by X-ray photon-electron spectrometry (XPS, Kα, Thermo, Waltham, MA, USA). Peaks of C1s between 280 and 292 eV was analyzed by software Magicplot (Magic systems, Saint Petersburg, Russia). The software is capable of removing background signals and iterative Gaussian/Lorentzian fitting.

### 2.7. In Vitro Cell Culture of BMSCs

Rat bone marrow stromal cells (BMSCs) harvested from 8-week-old Sprague-Dawley rats were cultured for determining the effect of PPy films on osteogenesis. These cells were maintained in DMEM with 10% FBS. Osteogenic supplement (100 μm ascorbic-2-phosphate, 10 mM β-glycerophosphate, and 100 nM dexamethasone) was applied to medium during the osteogenesis experiment.

### 2.8. Biocompatibility of PPy Films

To evaluate biocompatibility of PPy films, BMSCs were seeded to PPy films with density of 17,000 cells/cm^2^, and the (4,5-cimethylthiazol-2-yl)-2,5-diphenyl tetrazolium bromide (MTT) assay was used to quantify cell viability. After 2-day culture, 100 μL of MTT solution (5 mg/mL in PBS) and 900 μL of medium were added to each film and kept for 3 h at 37 °C. The supernatant was removed and 1 mL of dimethyl sulfoxide (DMSO) was added to dissolve formazan, which then was analyzed by the spectrometer at the wavelength of 550 nm.

### 2.9. Quantification of Calcium Deposition in Extracellular Matrix

The differentiated osteoblasts from BMSCs can deposit calcium in the extracellular matrix (ECM) as a process called mineralization [17]. To investigate this process, the calcium ortho–cresolphthalein complexone method (Ca-*o*-CPC) and alizarin red S staining were used to quantify the calcium deposition by BMSCs seeded on PPy films after 24 h.

The Ca-*o*-CPC assay measures the amount of calcium deposited in ECM by analyzing the optical absorbance of purple-colored complex at wavelength of 570/660 nm. The resulting increase in optical absorbance of the mixture is directly proportional to the calcium concentration in the solution via the following reaction [18]:

(1) $${\text{Ca}}^{2+}(\text{aq})+o-\text{CPC}(\text{aq})\to \text{Ca}-o-\text{CPC complex (violet)}$$

Before the assay, osteogenic medium was washed away from the well using PBS. Then, 100 μL of 0.5 N acetic acid was added to release calcium ions. Equal volume (200 μL) of calcium binding reagent (1 g/L of *o*-cresolphthalein complexone, and 0.1 g/L of 8-hydroxyquinoline) and calcium buffer reagent (1.6 M of 2-Amino-2-methyl-1-propanol, pH 10.7) were added to 10 μL of calcium released sample. After 15-min incubation at room temperature, 100 μL of purple-colored Ca-*o*-CPC complex was transferred to 96-well multiplates to measure its optical absorbance at wavelength of 575 nm. The amount of calcium in cell lysate was calculated according to the linear calibration results of calcium chloride standard solutions.

To normalize the amount of calcium deposition per cell, lactate dehydrogenase (LDH) assay was used to count the cell numbers using CytoTox 96 Non-Radioactive Cytotoxicity Assay (Promega, Madison, WI, USA). Briefly, 100 μL of fresh medium was replaced in each well before the assay, and 15 μL of lysis buffer was added to sample and kept for 1 h at 37 °C to release LDH from live cells. After transferring 50 μL of these LDH released medium to 96-well multiplates, 50 μL of LDH reagent was added and incubated for 30 min at room temperature. Finally, 50 μL of stop solution was added to each well and then were analyzed for the optical absorbance at wavelength of 490 nm. For calibration, a standard curve for lysing known cell numbers is applied to convert the optical absorbance to total cell number.

For alizarin red S staining, the cultures were rinsed with PBS followed by fixation (1% glutaraldehyde in PBS) for 30 min at 37 °C and then stained by 2% for alizarin red S solutions for another 20 minutes at room temperature. After PBS washes, the stained samples were examined by inverted microscope (Eclipse Ti-U, Nikon, Tokyo, Japan).

## 3. Results and Discussion

### 3.1. Characteristics of Microstructures and Chemical Composition

**Figure 1:**
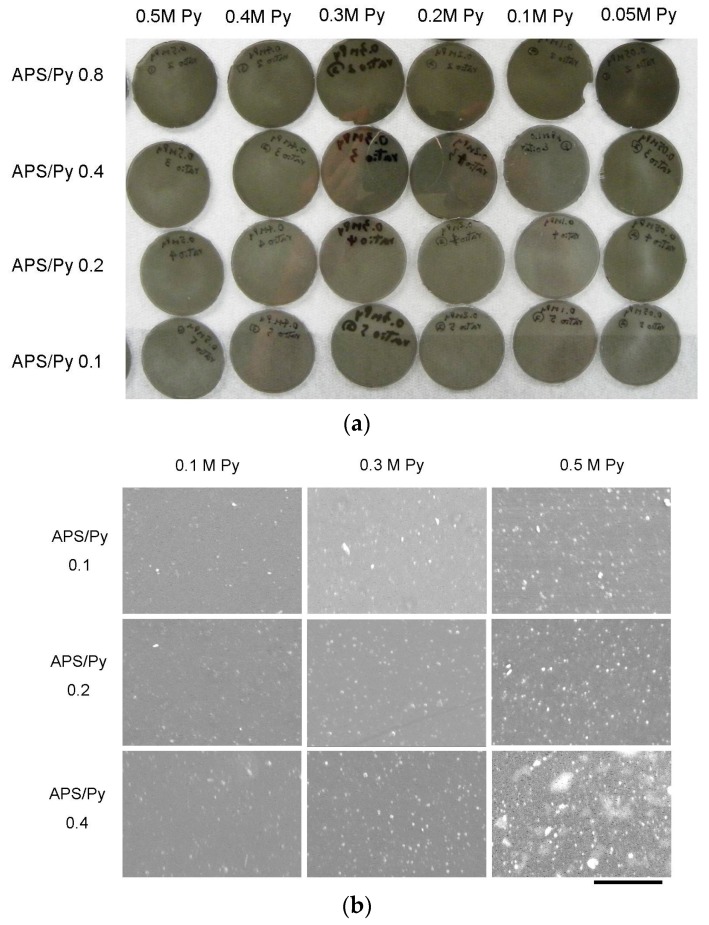

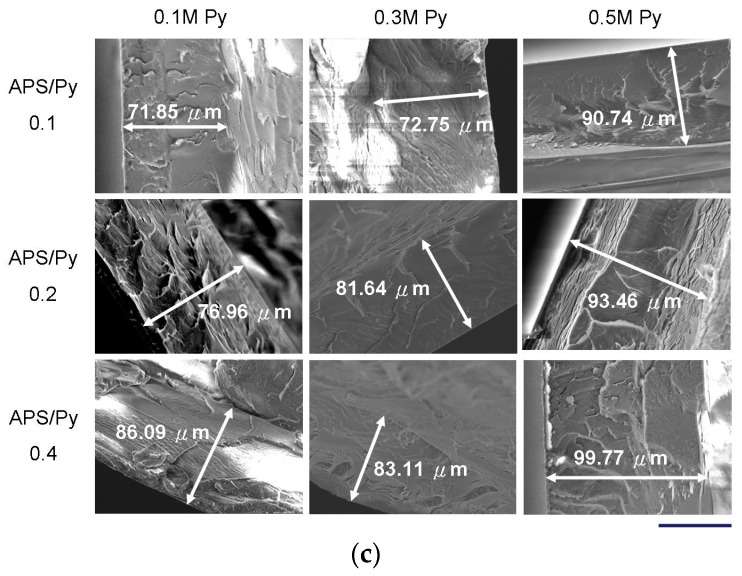
The fabricated polypyrrole (PPy) films: (**a**) PPy films were fabricated by different concentrations of pyrrole and APS/Py ratios; (**b**) surface roughness of PPy films by SEM (Scale bar = 10 μm); and (**c**) cross sections SEM of PPy films (Scale bar = 50 μm).

The synthesized PPy films with different APS to pyrrole concentration ratios (APS/Py) are shown in Figure 1a where all films are dark but transparent. Higher ratios of APS to Py lead to darker films. These films were also inspected by SEM. The roughness obviously increased with the pyrrole concentrations as well as APS/Py ratios, suggesting that the accelerated polymerization at high concentrations of monomer or initiator reduces the uniformity of films (Figure 1b). The cross section SEMs in Figure 1c show the dense structure of films and their thickness are estimated to be around 70 to 100 μm. Higher pyrrole concentration and APS/Py ratio tend to thicken PPy films slightly.

**Figure 2:**
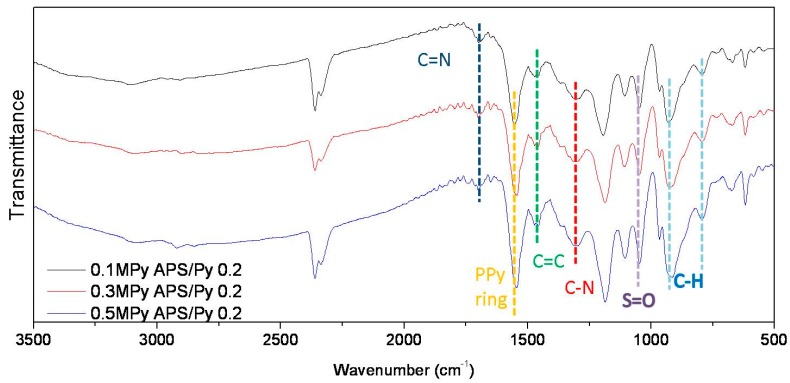
The FT-IR spectra of selected PPy films.

Figure 2 shows the ATR-FTIR analyses of selected PPy films. Similar spectrums are also obtained for other pyrrole concentrations. The two specific peaks at 1690 and 1360 cm^−1^ are ascribed to be C=N and C–N groups, respectively [19]. The absorptions at 1475 and 1550 cm^−1^ are due to stretch modes of C=C and pyrrole ring, respectively [20,21]. In addition, the absorption of S=O at 1050 cm^−1^ is attributed by the initiator APS [22]. These results confirmed that the fabricated PPy films were consistent with previous studies.

### 3.2. Electrical Resistance

**Figure 3:**
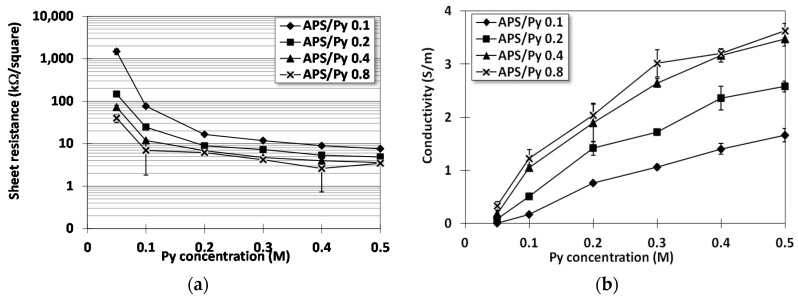
The electrical resistance of PPy films: (**a**) the sheet resistance; and (**b**) the conductivity (reciprocal of sheet resistance × film thickness).

Figure 3a presents the measured sheet resistivity of PPy films by the four point probe. It turns out that higher pyrrole concentrations and APS/Py ratios result in lower electrical resistance. However, notably, the sheet resistance of films fabricated using higher pyrrole concentration is much less affected by the ASP/Py ratio. In other words, the initiator has a limited influence on the electrical resistance of films if pyrrole concentration is high enough. Following the resistance measure, PPy films prepared by pyrrole concentrations of 0.1, 0.3, and 0.5 and APS/Py ratio of 0.1, 0.2, and 0.4 were used for the following study on cells. The corresponding conductivity, which is the reciprocal of sheet resistance × film thickness, is shown in Figure 3b for later data analyses.

Theoretically, APS/Py ratio should be 1:1 for ideal electron transfer. Blinova et al. have applied 0.2 M Py solution with different concentrations of APS, and their results suggested that 0.25 M of APS, i.e., APS/Py = 1.25, demonstrated the best conductivity [23]. However, their preparation was at acidic condition (with 0.2 M HCl). In this study, oxidation is performed at neutral condition, and the results suggest that film conductivity increased with increasing APS. However, conductivities of the APS/Py = 0.8 group is only slightly better than those of APS/Py = 0.4, suggesting the APS effect should be saturated. We deduce that Py in neutral form are not as easily oxidized as those in acidic condition.

### 3.3. XPS Analysis and Molecular Structures

**Figure 4:**
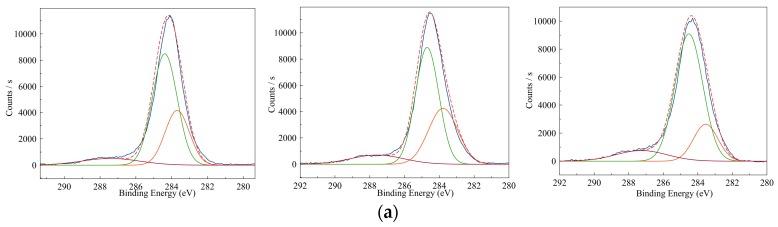

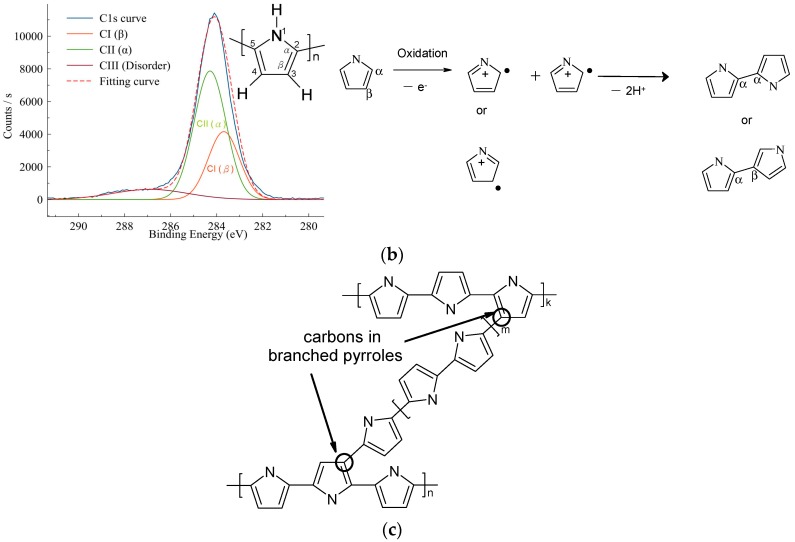
The chemical compositions of PPy films: (**a**) the XPS analysis; (**b**) schematic illustration of the cationic radicals of pyrrole link may each other through the 2,5 position (α–α’ linkage) or the 2,3 position (α–β’ linkage); and (**c**) the polymerization of pyrrole is mainly through the α–α’ linkage. However, if α–β’ linkage branches from the α–α’ linked main chains to form side chains or inter-chain links, the C1s of β carbons in branched pyrroles is shifted to form a disorder peak.

Figure 4a shows the XPS analyses on selected PPy films for the binding energy of C1s orbitals. In all analyses, the C1s peak can be de-convoluted by three Gaussian curves, namely CI(α), CII(β), and CIII(disorder) peaks, which corresponds to the α carbons, β carbons, and carbons at structural disorder sites, respectively (cf. Figure 4b) [24,25,26]. Pyrroles are polymerized via oxidation to form the pi-radical cation C_4_H_4_NH^+●^. These cationic radicals interact each other for radical-radical bond formation. This process can be repeated many times to form final PPy [23,27]. Following Pfluger’s study [25], the majority of the pyrrole units are linked through the C–2,5 carbons to form linear chains (α–α’ linkage). However, partial pyrroles can couple through the C–2,3 carbons (α–β’ linkages). If α–β’ linkage branches from the α–α’ linked main chains, it leads to side chain or inter-chain link formation (Figure 4c). Different from pyrroles in the α-α’ linked main chains, the hydrogen atoms on the β carbons of the branch pyrroles are changed to carbon atoms, which resulted in a disorder peak in a higher binding energy with larger width compared to α and β carbons. In this study, the electrophile attack or oxidation is carried out by the initiator APS. If high concentrations of monomer or initiator are introduced during polymerization, the chance of forming branched or cross-linked pyrroles would be also high and thus reduce the linear chain of PPy.

Therefore, the area ratios of disorder peak to sum of three peaks are defined as disorder ratios to quantify the extent of branching or crosslinking within PPy:

(2) $$\text{Disorder ratio}=\frac{\text{Area of CIII(disorder)}}{\text{Area of CI}(\alpha )+\text{Area of CII}(\beta )+\text{Area of CIII(disorder) }}$$

**Figure 5:**
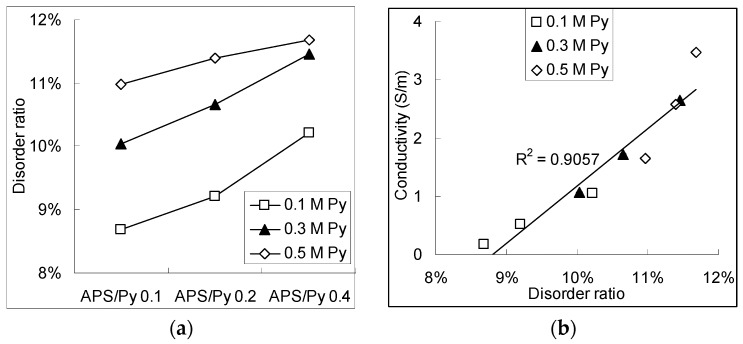
The chemical compositions of PPy films: (**a**) the branching ratio calculated for different APS/Py ratios in PPy films; and (**b**) the branching ratio as a function of the electrical conductivity of PPy films.

Figure 5a shows the disorder ratio as functions of the APS/Py ratio under different pyrrole concentrations. As the APS/Py ratio and pyrrole concentration becomes higher, the branching or crosslinking also shifts to high scores. The more branches and crosslinks there are, the more possible conducting paths for the electrons to move in the PPy film there will be. If we carefully correlated the electrical conductivity from Figure 3b with the disorder ratio, a linear function can be found in the Figure 5b. One possible 2D networking of PPy is schematically drawn in Figure 4c where the pi electrons can move or jump along many the inter-connection of pyrrole rings for conduction [24,25,26].

Similar effects of branching or crosslinking on conductivity have also been reported by Joo et al. [28]. Different from our study, they varied the dopant systems for chemical or electrochemical PPy synthesis. Their XPS results suggested that the levels of disorder of PPy are critical to PPy conductivity. PPy systems with lower interchain links or side chains resulted in weaker interchain interaction, which lead PPy in an insulating state. To explain this phenomenon, Prigodin et al. have developed a random network model of metallic wires, which indicated the concentration of the junctions, such as interchain linkage, highly impacts the insulator–metal transition in conducting polymers [29,30].

### 3.4. MTT Assay for the Biocompatibilities of PPy Films

**Figure 6:**
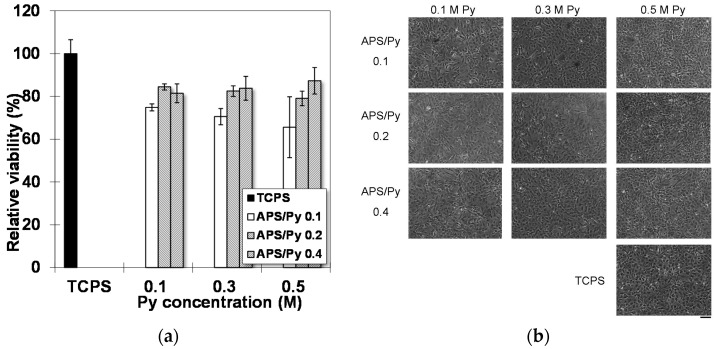
Biocompatibility of PPy films: (**a**) MTT assay; and (**b**) cell morphologies of BMSCs were observed one day after being seeded on PPy films (Scale bar = 100 μm).

MTT assay in Figure 6a shows that the average viabilities of rat BMSCs on different substrates. Compared to cells on tissue culture polystyrene (TCPS), the PPy films yield lower viabilities but still capable of maintaining it above 70%. Increasing APS/Py ratios significantly improved MTT results up to 80%~90%. This enhancement may be attributed to the good electrical conductivity of underlying PPy films, which can directly promote the cellular activities. On the other hand, the overall morphology of cells has no significant difference between cultured on TCPS and PPy films (Figure 6b). These results suggested that the PPy films are biocompatible.

### 3.5. Osteogenesis of BMSCs on PPy Films

**Figure 7:**
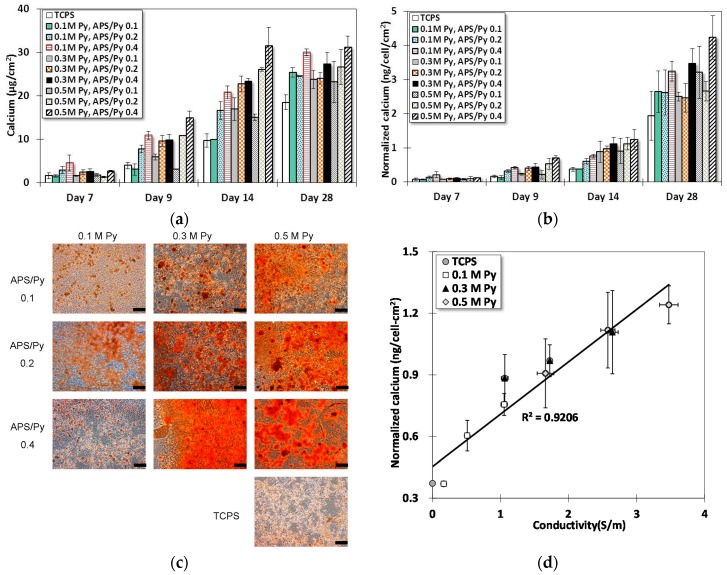
The calcium deposition of differentiated BMSCs on PPy films: (**a**) the calcium measured by the Ca-OCPC complex method; (**b**) the normalized calcium deposition in ECM using the surface cell number determined by the LDH assay; (**c**) alizarin red S staining shows the level of mineralization on Day 14 (Scale bar = 100 μm); and (**d**) the relation between normalized calcium deposition and electrical conductivity of PPY films.

Figure 7a shows the results from Ca-*o*-CPC assay for differentiated BMSCs on different substrates at different times. Note that there was no detectable calcium in all samples until Day 7. Starting from Day 7, the calcium content in all sample groups increases whether on TCPS or PPy films. Day 14 has the most significant increase among all sample groups. Films of APS/Py ratio 0.2 and 0.4 had differentiated BMSCs to boost the deposition of calcium faster than the groups of TCPS and lower APS/Py ratio 0.1. In particular, the films made of 0.5 M pyrrole and APS/Py ratio 0.4 have the most positive effects to promote the deposition of calcium into ECM, which almost tripled the amount of calcium by differentiated BMSCs on TCPS on Day 14. Another important remark is that the level of calcium deposition reached its saturation point at around 28 days. This observation may exemplify the required period for a full osteogenesis of BMSCs.

The quantity of calcium deposition can be normalized by the average number of cells (LDH assay) on the surface, as shown in Figure 7b. Although it seems no new information is provided by Figure 7b, as the plot presents a similar trend to Figure 7a, Figure 7b does clarify one possible doubt that the increase of calcium deposition is not primarily due to the proliferation (high cell numbers) but rather largely to the function of mineralization. Since differentiated cells can die in a mature bone matrix, the culture until 28 days does not necessarily have a high number of cells. Thus, the significantly higher calcium per unit area per cell on Day 28 evidently elucidates the suspicion.

Figure 7c presents the alizarin red S staining at Day 14. This is to confirm the results from the Ca-*o*-CPC assay. Obviously, the control group of TCPS, and the group of 0.1 M Py and APS/Py 0.1 have much less stained color than others. The higher pyrrole concentration and APS/Py ratio, the denser of the color exhibits in the microscopic photos. These are all in accordance with the results in Figure 7a.

The relation between normalized calcium concentration (Figure 7b) and electrical conductivity (Figure 3b) are plotted in Figure 7d where a linear regression fits the data fairly well ($R^{2}=0.9206$). This shows a strong support for the claim mentioned earlier that the electroactive surfaces can improve the osteogenesis of differentiated BMSCs (osteoblasts). One possible explanation for this correlation is that surface charges on these electroactive films facilitate the protein adsorption to induce a cascade of intra-cellular reactions for cell differentiation [31,32].

## 4. Conclusions

In this study, biocompatible PPy films are fabricated by oxidative chemical polymerization using different pyrrole concentrations and initiators to regulate their conductivities. These fabricated PPy films are transparent and can be used easily for optical microscopy. For the fabrication part, XPS together with four point probe show that increasing either the higher pyrrole concentration or monomer/initiator ratio can increase the branching ratio in PPy chains, which in turn facilitates the crosslinking or networking among monomers. The high crosslinks and networks definitely enhance the overall electron mobility and electrical conductivity. The highest electrical conductivity of films measured in this study is around 3.628 S/m for films made of 0.5 M pyrrole and APS/Py ratio at 0.8. For the biological part, bone marrow stromal cells of rats were chosen to cultivate on highly conductive PPy films. The MTT assay confirms the biocompatibility of fabricated films with viability more than 80% for APS/Py ratios of 0.2 and 0.4 in the PPy films. The Ca-*o*-CPC assay further demonstrates that on differentiated BMSCs (osteoblasts) on PPy films had enhanced calcium deposition or mineralization in ECM and reached the saturation after 28-day culture. Lastly, a good linear correlation between the electrical conductivity and calcium deposition by differentiated BMSCs on PPy films supported the proposed hypothesis, i.e., the electroactive surfaces of electrically conductive PPy films can promote the osteogenesis of cells.
